# Protocol for identifying and characterising critical physical tasks in the military: Development and validation

**DOI:** 10.3233/WOR-230263

**Published:** 2024-04-09

**Authors:** Elena Tseli, Andreas Monnier, Riccardo LoMartire, Linda Vixner, Björn Äng, Tony Bohman

**Affiliations:** a School of Health and Welfare, Dalarna University, Falun, Sweden; b Department of Neurobiology, Care Sciences and Society, Karolinska Institutet, Huddinge, Sweden; c Military Academy Karlberg, Swedish Armed Forces, Solna, Sweden; d Department of Research and Higher Education, The Administration of Regional Board, Center for Clinical Research Dalarna - Uppsala University, Falun, Sweden; e Department of Physical Education and Sport Sciences, Biomechanics and Ergonomics Laboratory, University of Thessaly, Trikala, Greece

**Keywords:** Evaluation study, military personnel, physical employment standards, questionnaire, work performance

## Abstract

**BACKGROUND:**

When establishing Physical Employment Standards, validity is dependent on the correct identification and characterisation of critical job tasks.

**OBJECTIVE:**

To develop and validate a standardised protocol for the identification, characterisation, and documentation of critical physical job tasks in military occupational specialities in the Swedish Armed Forces (SwAF), and propose a definition of critical physical job tasks for use in the SwAF.

**METHODS:**

A protocol was drafted with three content domains, including a preliminary definition. Protocol content validity was iteratively assessed in two consecutive stages where ten subject experts rated relevance and simplicity. A consensus panel revised the protocol after each stage. Content validity index (CVI) was calculated as item-CVI (I-CVI) per each feature and as scale average (S-CVI/Ave) per content domain. Acceptable content validity thresholds were 0.78 and 0.90, respectively.

**RESULTS:**

The validated protocol consisted of 35 items with an I-CVI≥0.90 and≥0.80 for relevance and simplicity, respectively. The S-CVI/Ave was 0.97 for relevance and 0.98 for simplicity. The protocol was language reviewed, reorganised for easy use, and approved by the consensus panel. The final protocol includes: background and aim of the protocol, the accepted generic and critical physical job task definitions, protocol instructions, subject expert-qualifications, job task source and characteristics.

**CONCLUSION:**

A standardised protocol for identification and characterisation of critical job tasks in SwAF military occupational specialties was developed. The protocol content was rated relevant and simple by experts and will be of importance in future work establishing physical requirements in the SwAF.

## 1 Introduction

Operational tasks in uniformed professions such as the military, fire service, and police can be physically demanding and can induce injury [1]. Physical Employment Standards (PES) are developed within these professions to ensure the effective and safe performance of operational tasks, while satisfying employment legislation [2–7]. PES aim to identify if an individual’s physical abilities meet the physical demands of a job or position, and therefore comprise the testing of physical capability derived from the job task that an individual performs [2, 6, 7].

Several well-established processes for the development of PES in uniformed services currently exist, but which one is preferable has not yet been established [1, 4]. However, consensus for a PES development guide in a military context was recently presented in a NATO Science and Technology Organization report [7]. The guide emphasises the importance of the initial process where a definition of a critical task is established, and that it is of great importance to adapt the established definition both to the organisational context and the country of origin. Once the definition of a critical task is established, five key steps in PES development are suggested in the NATO guidelines: 1) Job/Task Analysis, 2) Scenario Construction, 3) Test Development, 4) Setting Standards, and 5) Validation and Reliability.

The Job/Task analysis is the foundation on which a PES is developed. It involves identifying, documenting, and downselecting the critical tasks, using a combination of subjective and objective methods. Based on scenario constructions and ergonomic analyses, a final selection of critical tasks is conducted - which then informs the development of appropriate tests within a PES.

Most literature on PES development stresses the importance of defining, identifying, and characterising the critical tasks correctly early in the process [1, 7, 8]. This could be done using subjective and/or objective methods, but the infrequent and open-ended nature of many operational tasks in a military context, combined with the difficulties to quantify such tasks and the potential dangers associated with the observation of these tasks, most often requires the use of subjective methods during this phase [7]. Unfortunately, this process is often not fully reported in research [9]. It is also considered to be of the utmost importance for the validity of the PES development process that identification and characterisation of the tasks is performed by ‘subject matter experts’ with relevant knowledge and experience in the current context [1, 6–8, 10]. Furthermore, Tipton et al. highlight the importance of a clear audit trail concerning this early process of defining, identifying, and characterising the critical tasks, which is something that is missing in literature [6]. A clearly described audit trail would enhance the possibility of tracking information on the subjective methods used, and backtrack information sources, e.g. the subject matter experts (the users) involved and literature used, which would be essential for the quality of subsequent updates, and enhance the validity of the process.

Since 2019 the Swedish Armed Forces (SwAF) have initialised several projects aiming to develop and establish new physical requirements for military occupational specialties. To achieve this, the two initial steps in the PES development process, task analysis and scenario construction, first need completion. To aid the validity and reliability of the results from this part of the process, the SwAF decided to develop and validate a standardised protocol that may be used in the important first step of identifying and characterising critical tasks in different military occupational specialties. The resulting documentation can then be used as an important decision basis for upcoming physical capacity assessments that are to be included in further analysis and test development.

Several definitions of a critical physical job task have been proposed in PES literature [6–8, 11]. The most common proposition is to categorise critical physical job tasks into the subcategories “generic tasks” and “critical tasks” where the term “generic” defines a task performed on a regular basis that potentially could lead to undue stress to the individual or the group and “critical” defines a task that, regardless of frequency, if unsuccessfully performed, could be critical to the health and safety for the individual or group or endanger a mission, e.g. in an emergency situation.

The objective of this study was two-fold: (1) to develop and validate a standardised protocol for identifying, characterising, and documenting generic and critical physical job tasks in SwAF military occupational specialties, and (2) to propose a Swedish definition of critical physical job tasks for use in the SwAF.

## 2 Methods

### 2.1 Design and procedure

This content validity evaluation study was performed as a collaboration between Dalarna University, the SwAF, and the KTH Royal Institute of Technology. It is based on a protocol draft that aimed to identify critical physical job tasks in military occupational specialties and was constructed by the Military Academy Karlberg, SwAF Sports and Health Promotion Unit in 2019. Using this draft as a starting point and in accordance with recommendations, we further developed, validated, and completed a standardised protocol for identifying, characterising, and documenting critical physical job tasks in SwAF military occupational specialties [12–14]. The validation was performed using a systematic iterative process that included evaluation by subject experts, quantification, and revision by a consensus panel until determined valid. The study procedure was initiated in September 2019 and completed in June 2020.

### 2.2 Consensus panel and subject experts

The consensus panel included five of the authors of the present paper (ET, AM, RLM, BÄ, TB), an army officer of the SwAF, and a senior researcher from the KTH Royal Institute of Technology. The panel represented relevant areas of research and methodology such as epidemiology, questionnaire development and validation, musculoskeletal health, physical performance in a military context, and occupational and environmental physiology.

The subject experts were selected based on their experience and knowledge, striving for a balanced group composition with relevant and blended expertise [12, 13]. A convenience sample was used and, altogether, eleven subject experts volunteered for enrolment in the study including six men and five women (Table 1). The ranks of the military experts ranged from Lieutenant (OF1) to Captain (OF2) and First Sergeant (OR8). The experts signed a letter of consent including information concerning project background, their involvement in the validation process, and the possibility to withdraw at any time without giving reason. They were also informed that all personal data was to be handled in the strictest confidence.

**Table 1:** Participating subject experts, their field of expertise and profession

Expert	Expertise	Profession
1	Ergonomics, physical performance in the SwAF.	Research and Development Officer, Physiotherapist/Ergonomist, SwAF
2	Sport science, combat-readiness, war science, belongs to target group	Army Officer, Teacher Combat-Readiness, SwAF
3	Sport science, combat-readiness, formerly in target group	Army Officer, Course Coordinator/Instructor, Combat-Readiness, SwAF
4	Combat-readiness, physical capacity testing, belongs to target group	Naval Officer, Staff Administrator, SwAF
5	Extensive experience in target group	Army Officer, Platoon Commander, SwAF
6	Military education, belongs to target group	Army Officer, Platoon Commander, SwAF
7	Physical capacity testing, military training, belongs to target group	Naval Officer, Education Officer Naval Warfare, SwAF
8	Physiotherapy, ergonomics, belongs to target group	Naval Officer, Physiotherapist, SwAF
9	Physiology, environmental physiology, human research in military populations (SwAF)	Researcher, PhD, KTH Royal Institute of Technology
10	Occupational and environmental physiology, human research in military populations (SwAF)	Researcher, PhD, KTH Royal Institute of Technology
11	Physiotherapy, validation methods, medical sciences	Researcher, PhD, Dalarna University (author LV)

Abbreviations: SwAF: Swedish Armed Forces.

### 2.3 Development phase

An initial version of the protocol was developed by two of the authors (ET and TB) and was based on the earlier draft constructed by the SwAF Sports and Health Promotion Unit, Military Academy Karlberg. Support was provided by two representatives involved in the initial SwAF protocol draft (the SwAF consensus panel member (author AM) and subject expert #1). It was agreed that the protocol would include three content domains: A) background, aim of the protocol, and instructions to subject matter expert (targeted user of the protocol), B) information of the subject matter expert’s expertise, identified tasks, and task identification source, and C) characteristics of the identified task. Furthermore, a definition and categorisation of critical physical job tasks was formulated in Swedish and based on recent publications on PES development in a military context [6–8, 11]. The definition was discussed, iteratively revised, and eventually included in Domain A of the protocol. Further general decisions regarding the content and design of the protocol (as intended for the targeted users) agreed on were that: a) each targeted user, i.e. each subject matter expert, should identify five to ten critical physical job tasks pertaining to each subcategory; generic and critical respectively, b) the source of information should be traceable, e.g. the subject matter experts’ own experience of the task and literature used to identify the critical task, c) each item’s response should primarily be in multiple-choice format, and d) the protocol should be a general protocol that is possible to use in various military occupational specialties. The inclusion of source information was considered essential in order to ensure future traceability, i.e. an audit trail. The number of tasks to be identified by the subject matter expert (targeted user) (5–10 per subcategory) was considered feasible and at the same time sufficient to cover the most critical aspect of the majority of the SwAF military occupational specialties. The balance between data quality and respondent burden was carefully considered to minimise the number of items included. An item refers to any individual feature, for example a question or an explanatory or informative text, included in the protocol that was assessed by content validity ratings in the validation phase.

### 2.4 Validation phase

The validation phase was constituted by two iterative subject expert assessments with consensus panel revisions in between, and referred to as stage 1 and stage 2. The subject expert assesments were scheduled one week apart, for 3 hours each, with assessments lasting 1–1.5 hours. Subject experts assessed the relevance and simplicity of the individual items included in the validation tool, the overall relevance and simplicity of the content domains (A, B, and C), and of the entire protocol (D), using a four-point Likert rating-scale. The scale was quantified in accordance with recommendations for content validity assessment as presented in Table 2 [12, 14, 15].

**Table 2:** Rating framework for the assessment of relevance and simplicity of items, content domains, and the entire protocol

Relevance	Comments / improvement proposal
1. □ Very relevant (no revision needed)
2. □ Quite relevant (needs minor revision)
3. □ Somewhat relevant (needs major revision)
4. □ Not relevant
Simplicity	Comments / improvement proposal
1. □ Very simple (no revision needed)
2. □ Quite simple (needs minor revision)
3. □ Somewhat simple (needs major revision)
4. □ Not simple

Relevance referred to how relevant the items were in relation to the context. Simplicity referred to how well formulated and easy to read the items were, if they were easy to answer and if they were linguistically correct. In addition to rating relevance and simplicity, the experts were also asked to comment on their answers and make suggestions for improvements. Furthermore, following completion of a domain as well as at the end of the protocol, subject experts were instructed to make comments and suggestions for improvements in free text, for example on adding items, the length of the protocol, and their overall opinion of the protocol. A “control item”, which was clearly not relevant, was also included in the protocol in Stage 1 for validation purposes, with its aim being to check that the experts were participating actively and consciously [13].

Content validity of the protocol was quantified using two levels of the content validity index (CVI), item and scale, according to recommendations [12, 14, 15]. Item-level CVI (I-CVI) was calculated as the proportion of experts that provided a rating of either 1 (very relevant/very simple) or 2 (quite relevant/quite simple). We used a conservative calculation of I-CVI, i.e. dividing the number of experts rating an item 1 or 2 by the total number of experts providing ratings, even if expert ratings for the item were missing. Scale-level CVI average (S-CVI/Ave) was used to evaluate the relevance and simplicity of each content domain individually and the entire protocol, respectively. S-CVI/Ave was calculated by dividing the sum of the I-CVIs by the total number of I-CVIs. An item with I-CVI≥0.78 was considered to have “excellent” validity if it was rated by six to ten subject experts [12]. As an example, in a panel of 10 experts, 8 would have to rate a task as very relevant (1) or quite relevant (2) to reach a I-CVI of > 0.78, (8 of 10 = 0.80). Similiarly, the individual domains and the entire protocol were deemed excellent if S-CVI/ave was≥0.90 [12, 14].

In addition, the scale-level CVI universal agreement (S-CVI/UA) was calculated as the proportion of items rated 1 or 2 by *all* experts [15, 16]. The S-CVI/UA was used to evaluate the overall agreement between the I-CVI ratings by the experts, and is recommended as a complement to S-CVI/Ave. The S-CVI/UA was deemed ‘good’ if it was≥0.80 [15, 16].

After each assessment, the protocol was revised by the consensus panel according to the comments and ratings made by the experts. Items not reaching recommended levels for content validity were either removed or improved and kept for the next stage. Based on *a priori* decision, experts were excluded if they rated the relevance of the control question as 1 or 2 (i.e. relevant) and/or if their ratings showed considerable incongruence with the majority of experts [13, 14].

To fulfil the secondary aim of proposing a Swedish definition of critical physical job tasks for use in the SwAF, experts were presented with a suggested definition of critical physical job tasks, subcategorized into generic and critical physical job tasks, which was included in the protocol. In addition to rating relevance and simplicity, they were also asked to revise and improve the suggested definitions. The proposed definition including its two subacategories is presented in Swedish in the Supplementary material (here presented in English):

*Generic physical job task; A (physical) task within the military assignment that is performed recurrently (daily or several times/week), either on a regular basis or periodically, and that may lead to considerable physical burden to the individual(s) performing the task.*

*Critical physical job task; A (physical) task within the military assignment that if unsuccessfully performed could endanger a mission or could be critical to the health and safety of an individual, unit, or the public, or lead to considerable damage of equipment.*

### 2.5 Completion phase

Following the second, and final, validation stage, the protocol was revised into a practical format for the targeted user (subject matter expert), and suitable for the SwAF oncoming work, and the protocol was approved.

### 2.6 Ethical considerations

The study is exempt from the Swedish Ethical Review Act as the methods used do not affect participants physically or mentally, or infer an obvious risk of harm to participants physically or mentally. Furthermore, the study does not include sensitive personal data or information about criminal activity. Written informed consent was obtained from all participants (the subject experts) before the commencement of the study.

## 3 Results

**Fig. 1:**
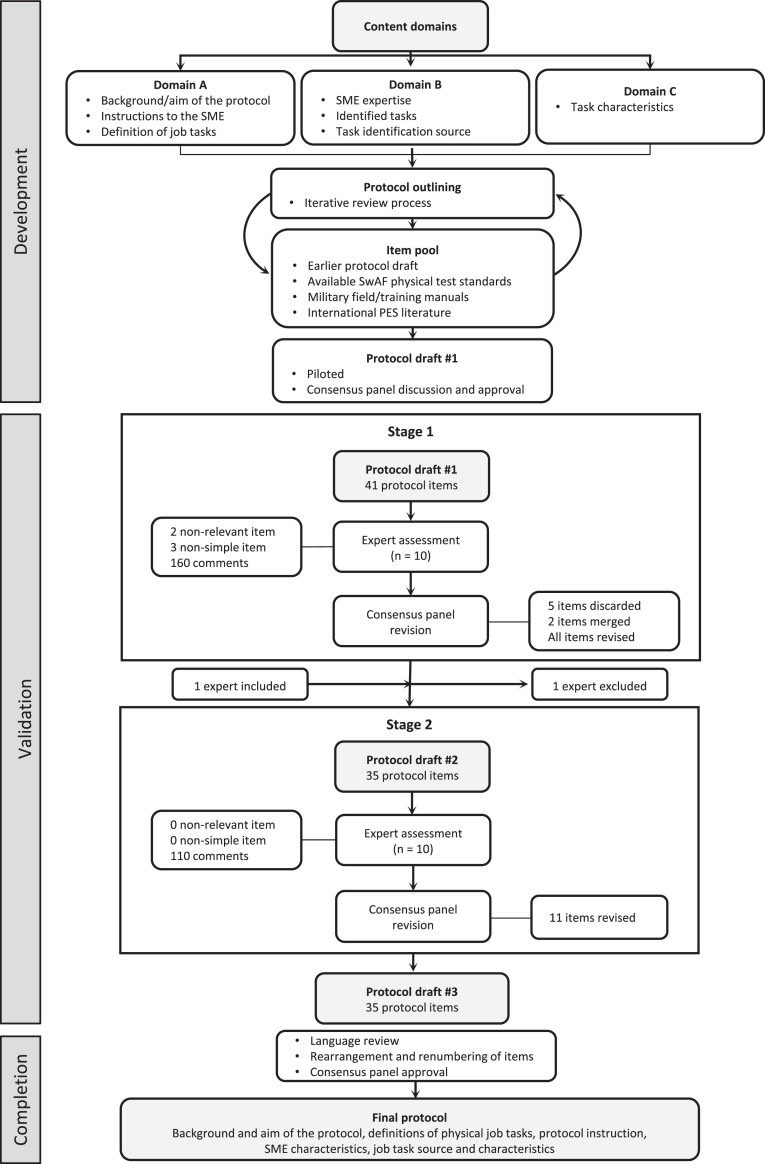
Flow chart of the study process including the three consecutive phases: development, validation, and completion. SME: Subject Matter Expert, SwAF: Swedish Armed Forces, PES: Physical Employment Standards.

An overview of the study process including the development, validation, and completion phases of the protocol is given in Fig.  1. The final protocol (in Swedish) is presented in the Supplementary material.

### 3.1 Development

The first protocol draft included three content domains: A) background and aim of the protocol, instruction for how to complete the protocol, and a suggested definition of a critical physical job task, consisting of two subcategories: a generic physical job task and a critical physical job task, a total of seven items. B) personal information of the subject matter expert (targeted protocol user), the military occupational specialties addressed, the identified critical physical job task, and the literary sources used (six items), and C) characteristics of the task; importance, performance and performer, environmental factors, equipment, frequency, duration, and manual handling (28 items) (Table 3). The choice of items included in Domain C was inspired by military field and training manuals, existing physical test standards in the SwAF, and reports on the development of PES in other countries [7, 8, 17–22] as well as descriptions concerning manual handling from the Swedish Work Environment Authority [23–26]. The draft was iteratively discussed and revised during the development phase, with the majority of revisions made in Domain C. This resulted in a protocol draft (#1) consisting of 41 items, where an item could represent a text paragraph or one, and in some cases two or three, multichoice questions. The protocol draft was then piloted by two of the authors (ET and TB) and four representatives from the SwAF. Finally, the draft was discussed and approved by the consensus panel. No further revision was considered necessary.

### 3.2 Validation

Table 3 shows the detailed information of item and scale level CVIs from both stages of the validitation process. The results are based on the ratings from 10 subject experts in each stage of the validation process, as one expert (#10) was unable to participate in stage 1 due to other work-related obligations and another (#11) was excluded in stage 2 due to the aforementioned exclusion criteria.

**Table 3:** Item-level content validity index and scale-level content validity index for the two assessment stages of the validation process

	Stage 1 (Protocol draft #1), (10 experts)					Stage 2 (Protocol draft #2), (10 experts)					
	Relevance		Simplicity		Relevance		Simplicity	
Domain	Item	Rating	I-CVI	Rating	I-CVI	Domain	Item	*(Item in stage 1)*	Rating	I-CVI	Rating	I-CVI
A. Background, instructions	A. Background, instructions
Background/aim for protocol	1	1–2	1.00	1–4	0.90	Background/aim for protocol	1	*(1)*	1–2*	0.90	1–3*	0.80
^^–″^–^	2	1–2	1.00	1	1.00	^^–″^–^	2	*(2)*	1–3	0.90	1–2	1.00
^^–″^–^	3	1–3	0.90	1–2*	0.90	^^–″^–^	3	*(3)*	1	1.00	1–2	1.00
Job task definition (generic)	4	1–2	1.00	1–3	0.90	Job task definition (generic)	4	*(4)*	1–2	1.00	1–3	0.90
Job task definition (critical)	5	1	1.00	1–3	0.90	Job task definition (critical)	5	*(5)*	1	1.00	1	1.00
Instructions for protocol	6	1–2	1.00	1–3	0.90	Instructions for protocol	6	*(6)*	1–3	0.90	1–3	0.90
^^–″^–^	7	1–2	1.00	1–2	1.00	^^–″^–^	7	*(7)*	1–4	0.90	1–2*	0.90
Domain A	A^a^	1–2	1.00	1–3	0.90	Domain A	A^a^	1	1.00	1–2	1.00
**S-CVI/Ave Domain A**			**0.99**		**0.93**	**S-CVI/Ave Domain A**				**0.94**		**0.93**
**B. SME, job task, source**	**B. SME, job task, source**
SME personal information	8	1–2	1.00	1–3	0.80	SME personal information	8	*(8)*	1–2	1.00	1	1.00
Identified job task	9	1	1.00	1–3	0.90	SwAF-position with the job task	9	*(11)*	1	1.00	1	1.00
Categorisation of job task	10	1–2	1.00	1–3	0.90	Identified job task	10	*(9)*	1	1.00	1–2	1.00
SwAF-position with the job task	11	1–3*	0.70	1–3	0.80	Categorisation of job task	11	*(10)*	1–2	1.00	1	1.00
SME job task experience	12	1–3	1.00	1–4	0.90	SME job task experience	12	*(12)*	1–2	1.00	1–2	1.00
Literature source	13	1–3	0.90	1–3	0.80	Literature source	13	*(13)*	1–2	1.00	1	1.00
Domain B	B^a^	1–2	1.00	1–3	0.90	Domain B	B^a^	1–2	1.00	1–2	1.00
**S-CVI/Ave Domain B**			**0.93**		**0.85**	**S-CVI/Ave Domain B**				**1.00**		**1.00**
**C. Job task characteristics**						**C. Job task characteristics**
Importance	14	1	1.00	1–3	0.90	Importance	14	*(14)*	1–3	0.90	1–2	1.00
Practitioners	15	1–2	1.00	1–4	0.70	Practitioners	15	*(15)*	1–2	1.00	1	1.00
Complexity	16^b^	1–4	0.90	1–4	0.70	Body position	16	*(17)*	1–2	1.00	1	1.00
Body position	17	1–4	0.90	1–4	0.80	Body motions	17	*(18)*	1–2	1.00	1–2	1.00
Body motions	18	1–4	0.90	1–4	0.80	Speed	18	*(19)*	1–3	0.90	1–2	1.00
Speed	19	1–4	0.90	1–4	0.80	Mandatory equipment	19	*(26)*	1–2	1.00	1–2	1.00
Most common environment	20	1	1.00	1–2	1.00	Additional equipment	20	*(28)*	1–2	1.00	1–2	1.00
Confined space	21	1–4	0.80	1–3	0.90	Frequency during service	21	*(29)*	1–2	1.00	1–2	1.00
Terrain	22	1–2	1.00	1–4	0.90	Frequency during education	22	*(30)*	1–2	1.00	1	1.00
Temperature	23	1–3	0.90	1–3	0.90	Duration during service	23	*(31)*	1–2	1.00	1–2	1.00
Time of year/day	24^c^	1–4	0.90	1–3	0.90	Duration during education	24	*(32)*	1–4	0.90	1–2	1.00
Additional factors of importance	25^c^	1–3	0.90	1–2	1.00	Type of manual handling if any	25	*(34)*	1	1.00	1	1.00
Mandatory equipment	26	1–2	1.00	1–3	0.90	Item to be handled	26	*(35)*	1–2	1.00	1–2	1.00
Control question	27^b^	1–4	0.20	1–4	0.50	Frequency of lifts	27	*(38)*	1–2	1.00	1	1.00
Additional equipment	28	1–3	0.90	1–4	0.90	Carrying distance	28	*(39)*	1–2	1.00	1	1.00
Frequency during service	29	1–2	1.00	1–4	0.80	Manual handling ergonomics	29	*(40)*	1–2	1.00	1	1.00
Frequency during education	30	1–2	1.00	1–4	0.80	Proportion of manual handling	30	*(41)*	1–3	0.90	1–3	0.90
Duration during service	31	1–2	1.00	1–4	0.80	Most common environment	31	*(20)*	1	1.00	1	1.00
Duration during education	32	1–2	1.00	1–4	0.90	Confined space	32	*(21)*	1–4	0.90	1	1.00
Importance of speed	33^b^	1–4	0.80	1–4	0.80	Terrain	33	*(22)*	1–2	1.00	1	1.00
Type of manual handling if any	34	1	1.00	1–2	1.00	Temperature	34	*(23)*	1–2	1.00	1	1.00
Item to be handled	35	1–2*	0.90	1–4	0.80	Light conditions/additional factors	35	*(24/25)*	1–2	1.00	1	1.00
Graspability of handled item	36^b^	1–4	0.90	1–4	0.90
Manual handling/body position	37^b^	1–3	0.90	1–4	0.90
Frequency of lifts	38	1–2	1.00	1–4	0.80
Carrying distance	39	1	1.00	1–3	0.90
Manual handling ergonomics	40	1–3	0.90	1–4	0.90
Proportion of manual handling	41	1	1.00	1	1.00
Domain C	C^a^	1–2	1.00	1–4	0.90		C^a^		1–2	1.00	1–2	1.00
**S-CVI/Ave Domain C**			**0.91**			**0.85**	**S-CVI/Ave Domain C**			**0.98**		**1.00**
Domain D Rating of entire protocol	D	1–2	**1.00**	1–3	**0.90**	Domain D Rating of entire protocol	D		1–2	**1.00**	1–2	**1.00**
**S-CVI/Ave Entire Protocol**			**0.93**		**0.87**	**S-CVI/Ave Entire Protocol**				**0.97**		**0.98**

^a^overall rating of the domain, ^b^discarded in stage 2, ^c^combined into one item in stage 2. Rating: 1 = very relevant/ very simple, 2 = quite relevant/ quite simple, 3 = somewhat relevant/ somewhat simple, 4 = not relevant/ not simple. I-CVI (item-level content validity index) = number of experts rating 1 or 2/total number of experts. S-CVI/Ave Domain (scale-level content validity index/average of the domain) = the average I-CVI across items in the domain. S-CVI/Ave Protocol (scale-level content validity index/average) = the average I-CVI across all items in the protocol. *One missing answer. Conservative calculation of I-CVI was used if answer was missing = number of experts rating 1 or 2/total number of experts, even if answers were missing.^–" –^ text paragraph, continued. **Abbreviations**: SME; Subject Matter Expert, SwAF-position; Position in the Swedish Armed Forces (SwAF).

After stage 2, the item “Background/aim for protocol” had an I-CVI of 0.80 for simplicity; in all the other items the I-CVI ranged from 0.90 to 1.00, indicating that all items reached “excellent” content validity (≥0.78, with 10 subject experts). Also the S-CVI/Ave indicated excellent validity in all domains and across all items in the protocol draft #2 (range from 0.93 to 1.00). All S-CVI/Ave either improved or stayed the same between stage 1 and 2, except for CVI/Ave Domain A for relevance which decreased from 0.99 to 0.94.

The S-CVI/UA, i.e. the proportion of items rated 1 or 2 by all experts, increased from 0.56 to 0.74 for relevance and from 0.15 to 0.86 for simplicity between stage 1 and stage 2, indicating that the overall agreement between the I-CVI ratings by the experts reached “good” agreement (≥0.80) for simplicity, but, despite being close, not for relevance.

Subject experts could also provide comments adjacent to the ratings. In stage 1, the majority of the 160 comments concerned simplicity. In stage 2, the number of comments was 110, mainly due to a reduction of comments in regard to simplicity. Additional notes (57 in stage 1 and 21 in stage 2) were also attained outside the allocated commentary fields. These were also included in the following consensus panel discussions.

Comments referred to the suggested definitions and concepts of military related constructs, linguistic clarifications, and suggestions for rephrasing. Moreover, comments concerned suggestions to increase usability within the target military occupational specialties. Although most ratings in stage 1 were above cut-off (≥0.78), practically all items were revised between protocol draft #1 (stage 1) and protocol draft #2 (stage 2) as a result of the comments and suggestions from subject experts (Table 3). Major changes included the exclusion of item 16 due to low simplicity and exclusion of items 33, 36 and 37 even though they reached recommended levels of I-CVI due to comments from the experts considering the overall understanding and importance. Moreover, a delimitation between the suitability of items for subjective assessment, through protocol or through objective measurements, affected the above mentioned changes. Items 24 and 25 were merged into one single item. Item 27 (control item) was also excluded as it had no purpose in stage 2. Finally, the order of some of the items were changed following recommendations from the experts. Thus, stage 1 resulted in the protocol draft #2 including a protocol with 35 items.

The second assessment stage resulted in protocol draft #3. Only minor revisions were deemed necessary by the consensus panel for protocol draft #3 (for 11 of 35 items). The consensus panel agreed that the protocol was sufficiently evaluated with regards to its content and decided that no further assessment round was needed.

#### 3.2.1 Proposed definition of critical physical job tasks including two subcategories: Generic and critical

The definitions of a generic physical job task reached an I-CVI of 1.0 for relevance and 0.9 for simplicity, and the definition of a critical physical job task reached an I-CVI of 1.0 for both relevance and simplicity (Table 3, items 4 and 5).

### 3.3 Completion

The consensus panel thoroughly revised the language and sorted item contents from the validated protocol draft #3 into individually numbered questions, in a format considered easy to follow for the subject-matter experts. The final protocol (Supplementary material) included: background and aim of the protocol, the final definitions of generic and critical physical job tasks, the instructions on how to complete the protocol (based on Table 3, items 1 to 7), and 63 questions covering subject matter experts’ characteristics, the identified job task, source and characteristics based on the remaining items (items 8 to 35) in protocol draft #3.

## 4 Discussion

In this study, we developed and validated a standardised protocol for identifying, characterising, and documenting critical physical job tasks in SwAF military occupational specialties. The protocol is structured in three content domains, A-C; where A provides the instructions and definitions, B details the expertise of the responding subject matter expert, the task in question, and the sources for its identification, and C details the characteristics of the specific task such as importance of the task, complexity, body position, speed, equipment, frequency of performance, environment etc. The protocol was determined to be valid with respect to its relevance and simplicity, with a S-CVI/Ave from 0.93 to 1.00 in all domains, and a S-CVI/Ave of 0.97 for relevance and 0.98 for simplicity for the entire protocol. Furthermore, we propose a validated definition of critical physical job tasks, generic and critical, for future use in the SwAF.

### 4.1 Methodological considerations

For a trustworthy content validation, even though the methodology and its analyses are quite straightforward, some components are considered essential.

*Identification of the content domain* was aided by the preliminary protocol draft, but still required a thorough review of relevant literature on the topic, in this case military context and ergonomic analyses, to operationalize the concept with adequate content. We believe the substantial preparations in this initial phase allowed for high ratings of content relevance already at the first assessment stage.

*Careful selection of content experts*, to achieve adequate comprehensiveness of knowledge and experience [13, 27]. Experts were carefully selected to cover important areas of information and included officers experienced in the military context as representatives of the target population. To fulfil our objectives, we also aimed for our expert group to include other topics of expertise, such as occupational and environmental physiology and questionnaire methodology and thereby experts’ content capability becomes increasingly heterogenous. This may impact on the ratings of some items, as all experts might not have relevant knowledge regarding all of them. Based on previous experiences within our author group [28–30] and recommendations [13, 14] we set up criteria for exclusion of subject experts in advance, which proved helpful as we excluded one of the experts in stage 2.

We provided information on the purpose of the study, operational definitions, and the rating task, to *maximize the assessment* in a joint start-up meeting [12, 27].

We included a *sufficient number of participants* to minimize the risk of chance agreement, and applied the most frequently used cut-off scores for acceptable content validity in line with Polit et al., 2007 [14]. We consistently used conservative calculations to avoid overestimation of agreement, still the CVI-ratings all showed excellent validity. All in all, we trust the methodology was appropriate to address our objectives. Even so, the content validity could be limited by the competence of the consensus panel and the experts.

### 4.2 Discussion of the results

We used a systematic, iterative development and evaluation process, combined with CVI calculations to establish the content validity of a proposed protocol. Similar methodology has previously been successfully used by our research group in various contexts for validation processes in instrument development [28, 29, 31] as well as intervention development [30, 32]. We perceive it as a practical, scientific approach to ascertain fundamental aspects of validity, like relevance, comprehensiveness and simplicity/comprehensiblity, when developing new instruments such as the current protocol.

Surveys and questionnaires constitute recommended subjective tools for job task analysis [7], however, for job task identification, information is often gathered from expert opinions, such as focus groups, by literature review or direct observations [7, 22]. These methods, while quite resource demanding, still entail limited ability to cover all military contexts and situations. Furthermore, if surveys are used as part of the task identification phase, they are often designed for that specific group of military occupational specialty [33] or service branch [19]. In contrast, this protocol was intended to be a standardised generic method for broad use in the task inventory phase within a military context. As such, this protocol has the potential to be an important asset in the early phase of PES construction; to identify and collect all necessary data upon which to base a foundation for the standards. It can be widely distributed and completed on a local basis, by subject matter experts with relevant knowledge and experience in the current context, which provides rich data yet requires fewer resources.

The protocol uses a simple format for the collection of comparable data, with Domains B and C repeated for every identified task, resulting in 5–10, characterised, and consistently documented job tasks for generic and critical physical job tasks, respectively. The three content domains A –C serve different purposes; Domain A, with instructions and the definition of critical physical job tasks, sets a common understanding of the purpose and procedure of the task identification when the protocol is applied locally within diffferent MOS. Domain B details the subject matter expert’s field of expertise, the job task and information source. This “audit trail” provides a unique possibility to backtrack the subject matter experts and sources, which could be of importance in a changing context as military physical job tasks change and evolve over time. Finally, Domain C details the job task characteristics, providing an information base on the various conditions and demands that the job task poses on the individual with regard to extrinsic and intrinsic factors, which will guide the selection of obejctive measurments to follow in next step.

The NATO Science and Technology Organization Report 2019 on combat integration and the implications for PES points to the prevalent musculoskeletal injuries among military personnel, the importance of identifying critical job tasks and the role of PES in musculoskeletal injury prevention [7]. Therefore, we believe this protocol will be an effective tool for a methodical task inventory phase to guide ongoing work with development of PES in the SwAF, while ensuring the traceability of information sources used in the process. Moreover the standardized documentation of the critical physical job tasks will inform how to best perform the following inventory of physical requirements and provide guidance on what objective measures will be required.

Before putting it into effect in SwAF, we recommend the protocol first be piloted on a small scale to evaluate practical dimensions such as the time required to complete the protocol, handling of the task lists, and other feasibility aspects of importance for successful implementation in real-life military context, in line with current recommendations [34]. Moreover, a web-based protocol could further increase its practicality and effectiveness. The generic structure and content of the protocol may also be of value for the inventory of critical tasks and physical requirements in other tactical populations. Our systematic approach for validating its relevance for the target popluation may inspire future applications in more generalised contexts.

## 5 Conclusion

A standardised protocol for identification and characterisation of critical job tasks in SwAF military occupational specialties was developed. The iterative validation process enabled careful improvements of the protocol, with revisions made in close collaboration with stakeholders. The protocol content was determined to be relevant and simple by experts and will be of importance in future work establishing physical requirements in the SwAF.

## Ethical approval

Not applicable.

## Informed consent

Before the study started, written informed consent was obtained from each participant (the subject experts).

## Conflict of interest

The authors declare that they have no conflict of interest.

## Supplementary Material

Supplementary Material

## References

[1] Nevola VR , Lowe MD , Marston CA . Review of methods to identify the critical job-tasks undertaken by the emergency services. Work. 2019;63(4):521–36. 31033477 10.3233/WOR-192914 PMC6839475

[2] Jamnik V , Gumienak R , Gledhill N . Developing legally defensible physiological employment standards for prominent physically demanding public safety occupations: a Canadian perspective. European Journal of Applied Physiology. 2013;113(10):2447–57. 23494548 10.1007/s00421-013-2603-1

[3] Payne W , Harvey J . A framework for the design and development of physical employment tests and standards. Ergonomics. 2010;53(7):858–71. 20582767 10.1080/00140139.2010.489964

[4] Petersen SR , Anderson GS , Tipton MJ , Docherty D , Graham TE , Sharkey BJ , et al. Towards best practice in physical and physiological employment standards. Appl Physiol Nutr Metab. 2016;41(6 Suppl 2):S47–62. 27277567 10.1139/apnm-2016-0003

[5] Taylor NA , Fullagar HH , Mott BJ , Sampson JA , Groeller H . Employment Standards for Australian Urban Firefighters: Part The Essential, Physically Demanding Tasks. J Occup Environ Med. 2015;57(10):1063–71. 26461861 10.1097/JOM.0000000000000525

[6] Tipton MJ , Milligan GS , Reilly TJ . Physiological employment standards I. Occupational fitness standards: objectively subjective? Eur J Appl Physiol. 2013;113(10):2435–46. 23263741 10.1007/s00421-012-2569-4

[7] NATO STrCombat Integration: Implications for Physical Employment Standards. Technical Report 2019 12/5/2019. Report No.: STO-TR-HFM-269.

[8] Reilly TJ , Gebhardt DL , Billing DC , Greeves JP , Sharp MA . Development and Implementation of Evidence-Based Physical Employment Standards: Key Challenges in the Military Context. J Strength Cond Res. 2015;29(Suppl 11):S28–33. 26506194 10.1519/JSC.0000000000001105

[9] Beck B , Billing DC , Carr AJ . Developing physical and physiological employment standards: Translation of job analysis findings to assessments and performance standards –A systematic review. International Journal of Industrial Ergonomics. 2016;56, 9–16.

[10] Blacklock RE , Reilly TJ , Spivock M , Newton PS , Olinek SM . Standard Establishment Through Scenarios (SETS): A new technique for occupational fitness standards. Work. 2015;52(2):375–83. 26409372 10.3233/WOR-152128

[11] Milligan GS , Reilly TJ , Zumbo BD , Tipton MJ . Validity and reliability of physical employment standards. Appl Physiol Nutr Metab. 2016;41(6 Suppl 2):S83–91. 27277570 10.1139/apnm-2015-0669

[12] Lynn MR . Determination and quantification of content validity. Nurs Res. 1986;35(6):382–5. 3640358

[13] Grant JS , Davis LL . Selection and use of content experts for instrument development. Research in Nursing & Health. 1997;20(3):269–74. 9179180 10.1002/(sici)1098-240x(199706)20:3<269::aid-nur9>3.0.co;2-g

[14] Polit DF , Beck CT , Owen SV . Is the CVI an acceptable indicator of content validity? Appraisal and recommendations. Res Nurs Health. 2007;30(4):459–67. 17654487 10.1002/nur.20199

[15] Polit DF , Beck CT . The content validity index: are you sure you know what’s being reported? Critique and recommendations. Res Nurs Health. 2006;29(5):489–97. 16977646 10.1002/nur.20147

[16] Hambleton RK , Swaminathan H , Algina J , Coulson DB . Criterion-Referenced Testing and Measurement: A Review of Technical Issues and Developments. Review of Educational Research. 1978;48(1):1–47.

[17] Bergh UD , Ekblom, U. Ö , et al. Fysiska prov för officerare –Utveckling av FM Fysisk Standard. 2005. Contract No.: FOI-R—-SE.

[18] Bergh UE , Engström Ö. L-M , et al. Försvarsmaktens Fysiska Standard –försök med fysisk träning som arbetsuppgift. 2008. Contract No.: FOI-R—-SE.

[19] Brown PEH , Fallowfield JL . Physical Employment Standards for UK Royal Navy Personnel: A Survey of Tasks That Require Muscle Strength and Endurance. Mil Med. 2019;184(11-12):882–8. 31067314 10.1093/milmed/usz099

[20] Regler för Försvarsmaktens Fysiska Standard, (2016).

[21] Försvarsmaktens Fysiska Standard –FM FysS, (2019).

[22] Spivock M , Reilly T , Newton PS , Blacklock RE , Jaenen S Project FORCE Phase I Report: Identification of common, essential, physically demanding tasks in the CF., Department of National Defence ADMSa, Ottawa. T; 2011.

[23] Arbetsmiljöverket Bedöm risker vid manuell hantering –skjuta/dra,KIM2 2012 [Available from: https://www.av.se/arbetsmiljoarbete-och-inspektioner/publikationer/broschyrer/bedom-risker-vid-manuell-hantering—skjutadra-adi668-broschyr/?hl=kim%201.

[24] Arbetsmiljöverket Bedöm risker vid manuell hantering - lyfta och bära,KIM1 2012 [Available from: https://www.av.se/arbetsmiljoarbete-och-inspektioner/publikationer/broschyrer/bedom-risker-vid-manuell-hantering—lyftabara-adi-627-broschyr/?hl=kim%201.

[25] Arbetsmiljöverket Riskbedömning av repetitivt arbete med stöd av nyckelindikatorer,KIM3 2012 [Available from: https://www.av.se/globalassets/filer/checklistor/riskbedomning-repetitivt-arbete-kim-3-checklista.pdf?hl=kim%203.

[26] Arbetsmiljöverket Arbetsmiljöverkets föreskrifter och allmänna råd om belastningsergonomi, AFS 2012:2 2012 [Available from: https://www.av.se/arbetsmiljoarbete-ochinspektioner/publikationer/foreskrifter/belastningsergonomi-afs-20122-foreskrifter/.

[27] Davis LL . Instrument review: Getting the most from a panel of experts. Appl Nurs Res. 1992;5(4):194–7.

[28] de Alwis MP , Lo Martire R , Ang BO , Garme K . Development and validation of a web-based questionnaire for surveying the health and working conditions of high-performance marine craft populations. BMJ Open. 2016;6(6):e011681. 10.1136/bmjopen-2016-011681 PMC4916626 27324717

[29] Lo Martire R , de Alwis MP , Äng BO , Garme K . Construction of a web-based questionnaire for longitudinal investigation of work exposure, musculoskeletal pain and performance impairments in high-performance marine craft populations. BMJ Open. 2017;7(7). 10.1136/bmjopen-2017-016006 PMC5642765 28729320

[30] Westman A , Äng BO . Validation of a free fall acrobatics intervention protocol to reduce neck loads during parachute opening shock. BMJ Open Sport & Exercise Medicine. 2015;1(1). 10.1136/bmjsem-2015-000045 PMC5117032 27900113

[31] Nilsson J , Friden C , Buren V , Ang BO . Development and validation of a web-based questionnaire for surveying skydivers. Aviat Space Environ Med. 2011;82(6):610–4. 21702311 10.3357/asem.2966.2011

[32] Tseli E , Sjoberg V , Bjork M , Ang BO , Vixner L . Evaluation of content validity and feasibility of the eVISualisation of physical activity and pain (eVIS) intervention for patients with chronic pain participating in interdisciplinary pain rehabilitation programs. PLoS One. 2023;18(3):e0282780. 36897847 10.1371/journal.pone.0282780 PMC10004540

[33] Larsson J , Dencker M , Olsson MC , Bremander A . Development and application of a questionnaire to self-rate physical work demands for ground combat soldiers. Appl Ergon. 2020;83, 103002. 31747636 10.1016/j.apergo.2019.103002

[34] Eldridge SM , Lancaster GA , Campbell MJ , Thabane L , Hopewell S , Coleman CL , et al. Defining Feasibility and Pilot Studies in Preparation for Randomised Controlled Trials: Development of a Conceptual Framework. PLOS ONE. 2016;11(3):e0150205. 26978655 10.1371/journal.pone.0150205 PMC4792418

