# Supplementary material for: Protocol for identifying and characterising critical physical tasks in the military: Development and validation
Source: Work. 2024 Apr 9;77(4):1261–72. doi: 10.3233/WOR-230263 (PMC11091656; doi:10.3233/WOR-230263)
Supplement: Supplementary Material [file wor-77-wor230263-s001.docx]

## PROTOCOL FOR THE IDENTIFICATION OF PHYSICAL TASKS WITHIN MILITARY ASSIGNMENTS

**Contents**

Background and purpose 2

Definitions 3

Instructions for completing the protocol 3

Parts of the protocol 4

Part 1. Information about you. 4

Part 2. Military assignment and physical tasks that are identified. 6

Part 3. Your experience of the physical task 8

Part 4. Sources/basis for the identification/description of the physical task. 9

Part 5. The nature of the physical task 10

5.1 The significance of the physical task for the military assignment 10

5.2 The individuals in the military assignment who perform the physical task 10

5.3 Execution of the physical task 11

5.4 Equipment 14

5.5 Frequency at which the physical task is performed in its entirety 16

5.6 Duration for which the physical task is performed in its entirety 18

5.7 Manual handling 20

## Background and purpose

| The Swedish Armed Forces have the task of establishing threshold values regarding the physical capacity that corresponds to the requirements of military assignments. These threshold values can then be used to establish so-called Physical Employment Standards and/or as guidelines for training physical capacity during education programmes for various military assignments.  In order to establish these threshold values, the Swedish Armed Forces need to conduct a physical task requirement analysis. Such an analysis aims to determine the physical capacity required to perform the tasks included in a military assignment efficiently and safely.  A crucial initial step, vital for the validity of the job requirement analysis, is to identify, from a physical perspective, the ‘Generic’ and ‘Critical’ tasks (see definitions below) that need to be capacity-determined.  The tasks can range from daily physical tasks to tasks that may never be required, but if they are they should be able to be performed satisfactorily and safely. |
| --- |
| The purpose of this protocol is therefore to **identify these Generic and Critical physical tasks** and to **document the characteristics of the tasks,** for example how often and how long a task is performed for, etc.  The protocol **should also include information about** the **sources/bases** used to identify and characterise the tasks, such as regulations, doctrines, manuals, assignment instructions, etc. |
| The identification of these physical tasks should be carried out with the assistance of individuals who have significant experience and/or understanding of physical tasks within their respective military assignments. These individuals are known as “Subject Matter Experts” (SMEs).  You have been asked to assist in the identification and description of these generic and critical physical job tasks, as you have been deemed to meet the requirements of a SME within your current military assignment. Your answers will provide important information for this analysis of physical requirements. |

##

## Definitions

| **Generic physical job task** | A (physical) task within the military assignment that is performed recurrently (daily or several times/week), either on a regular basis or periodically, and that may lead to considerable physical burden to the individual(s) performing the task. |
| --- | --- |
| **Critical physical job task** | A (physical) task within the military assignment that if unsuccessfully performed could endanger a mission or could be critical to the health and safety of an individual, unit, or the public, or lead to considerable damage of equipment. |

## Instructions for completing the protocol

| With help from the protocol, you are asked to **identify at least 5, but preferably 10, generic physical tasks and at least 5, but preferably 10, critical physical tasks in your current military assignment**. The tasks do not need to be ranked.  **Do not include physical training or physical tests.**  The protocol consists of five parts:   1. Information about you. 2. Military assignment and physical tasks that are identified. 3. Your experience of the physical tasks. 4. Sources/basis for the identification/description of the physical task. 5. The nature of the physical task. |
| --- |
| **Start by filling out this protocol with information about the first physical task you have identified. Then use a copy of Appendix 1 for each additional task you identify.** |
| If you cannot identify **at least 5** generic or critical physical tasks, please explain why here. |

## Parts of the protocol

### Part 1. Information about you.

| **1** | First name and last name |  |
| --- | --- | --- |
| **2** | Year you were born |  |
| **3** | Gender | Female  Male |
| **4** | Number of years in the Swedish Armed Forces  *Note. Please include basic training/military service.* | 0 to 2  >2 to 4  >4 to 6  >6 to 8  >8 to 10  >10 to 12  >12 to 14 >14 to 16  >16 |
| **5** | Education background in the Swedish Armed Forces  *E.g. officer training, civilian education, instructor courses, etc.*  *Specify the year for each education programme.* |  |
| **6** | Current military assignment in the Swedish Armed Forces. |  |
| **7** | Number of years in current assignment. | 0 to 2  >2 to 4  >4 to 6  >6 to 8 ☐ >8 to 10 ☐ >10 to 12  >12 to 14 ☐>14 to 16 ☐ >16 |
| **8** | Specify where and at which military unit you serve (your peacetime assignment). |  |
| **9** | Specify any previous positions you have held in the Swedish Armed Forces. |  |

###

### Part 2. Military assignment and physical tasks that are identified.

| **10** | Specify the military assignment in which the identified physical task is included. |  |
| --- | --- | --- |
| **11** | Is the physical task included in military assignments other than above? | No  Yes  Unsure  If ”Yes” specify other examples of military assignments that include the physical task;  _____________________________________  _____________________________________ |
| **12** | For which physical task does this protocol apply?  *Specify the task with a specific name if applicable, or name it in your own words.* |  |
| **13** | Describe the execution of the physical task. |  |
| **14** | What type of physical task is it?  *Check the box that applies.* | Generic physical task  *A (physical) task within the military assignment that is performed recurrently (daily or several times/week), either on a regular basis or periodically, and that may lead to considerable physical burden to the individual(s) performing the task.*  Critical physical task  *A (physical) task within the military assignment that if unsuccessfully performed could endanger a mission or could be critical to the health and safety of an individual, unit, or the public, or lead to considerable damage of equipment.*  Both options apply to the task. |
| **15** | The physical task is performed …  *Check both alternatives if necessary.* | ..…during the execution of the military assignment  ..…during training for the military assignment. |

###

### Part 3. Your experience of the physical task

| **16** | Assess your experience of the identified physical task in this protocol by responding to the following statements.  *Please respond to all 9 statements.* | 1. I have experience of performing the task during a military assignment or training.  *Yes*  *No*  2. I have experience of performing the task during national operations.  *Yes*  *No*  3. I have experience of performing the task during international operations.  *Yes*  *No*  4. I have experience of performing the task during an emergency situation.  *Yes ☐ No*  5. I have experience in a leadership position of instructing subordinates to perform the task and observing them as they do so.  *Yes ☐ No*  6. I have seen the task being performed in a satisfactory manner.  *Yes*  *No*  7. I have seen the task fail and can testify to the reasons for and the consequences of this failure (*for example, a soldier not being strong enough to drag an injured person to safety, requiring another soldier to assist and therefore not being able to complete their own task).*  *Yes ☐ No*  8. I have seen the task performed and/or performed the task myself in different ways and can assess the advantages and disadvantages of the various ways that the task can be performed.  *Yes ☐ No*  9. I have experience in providing formal training of the task (such as courses, development of training materials, etc.)  *Yes ☐ No* |
| --- | --- | --- |

### Part 4. Sources/basis for the identification/description of the physical task.

| **17** | What sources have been used to identify and describe the physical activity?  *Check more than one alternative if necessary.* | A. Literature *(for example: regulations, doctrines, manuals, assignment instructions, assignment descriptions, unit handbook)*  B. My experience of performing the task myself  C. My own experience from seeing the task being performed |
| --- | --- | --- |
| **18** | Specify the literature you have used in as much detail as possible.  *E.g. title, year published, relevant pages where task is mentioned/described, etc.* |  |

###

### Part 5. The nature of the physical task

#### 5.1 The significance of the physical task for the military assignment

|  | Estimate the **significance** (importance) of the identified physical task **for the military assignment.** | |
| --- | --- | --- |
| **19** | How significant/important is the physical task **for the military assignment**? | Very significant/important  *(e.g. it is not possible to complete the military assignment without performing the physical task)*  Significant/important  Quite significant/important  Not so significant/important  *(e.g. the physical task may not be needed to be performed in the military assignment)* |
| **20** | Explain your answer to the question above. |  |

#### 5.2 The individuals in the military assignment who perform the physical task

| **21** | The physical task **should be able to be performed**...  *More than one alternative is possible.* | …alone  …in a group  …sometimes alone and sometimes in a group |
| --- | --- | --- |
| **22** | If/when the task is performed in a group, how many people are needed to perform the physical task?  *More than one alternative is possible.* | 2–3  4–5  6–7  8–10  >10  The task is not performed in a group |

#### 5.3 Execution of the physical task

| **23** | In which position(s) is the physical task **usually** performed?  *Check more than one alternative if necessary.* | Standing  Sitting  Lying on back  Lying on front  Kneeling/one knee  Kneeling/two knees  Crouching  Other position?  _____________________________________  None of the above as the task is performed while moving | | |
| --- | --- | --- | --- | --- |
| **24** | In which position(s) **should** the physical task **be able to be** performed?  *Check more than one alternative if necessary.* | Standing  Sitting  Lying on back  Lying on front  Kneeling/one knee  Kneeling/two knees  Crouching  Other position?  _____________________________________  None of the above as the task is performed while moving | | |
| **25** | Which movements are **usually required** for the individual to be able to perform the physical task? | Walking     Crawling  Climbing  Bending forwards | | Running  Belly crawling  Deep squats  Crouching down |
|  |  | Swimming  Skiing  Reaching for something to the front or side  Reaching for something above shoulder height  Bending down forwards with straight/almost straight legs  Twisting the torso to the left or right | | |
| **26** | If movements other than those listed above are used in the physical task, please describe them here | I have no other movements to describe. | | |
| **27** | Which movements **must the individual to be able to do** so they can perform the physical task? | Walking  Crawling  Climbing  Bending forwards | Running  Belly crawling  Deep squats  Crouching down | |
|  |  | Swimming  Skiing  Reaching for something, to the front or side  Reaching for something above shoulder height  Bending down forwards with straight/almost straight legs  Twisting the torso to the left or right | | |
| **28** | If movements other than those listed above are required in order to perform the physical task, please describe them here | I have no other movements to describe. | | |
| **29** | At what speed is the physical task **usually** perfumed? | Slowly  Quite fast  Very fast  As fast as possible | | |
| **30** | At what speed **should** the physical task **be able to be** performed? | Slowly  Quite fast  Very fast  As fast as possible | | |

####

#### 5.4 Equipment

| **31** | Which of the following equipment **should be used** when performing the physical task.  *Any additional equipment is addressed in the next question.*  *Note: The uniform or work attire may vary, such as naval combat suit or M90, but the difference is marginal, so we choose to refer to it simply as uniform or work attire.* | Uniform/work attire for indoor tasks  Uniform/work attire for outdoor tasks  Flight suit for aviation duty  Helmet  Helmet for aviation duty  Helmet for vehicle-related tasks (snowmobile, motorcycle, combat vehicle)  Combat vest (alt. ”combat harness with pockets” alt. pockets attached to body armour)  Personal weapon (specify which one(s))  ___________________________________  Protective equipment for smoke diving, firefighting or rescue operations  Body armour without reinforced plates  Body armour with reinforced plates  Combat backpack (35 litres, packed according to the packing plan in the *Personal Equipment Manual* 2019),  Combat backpack 2000 or 12 |
| --- | --- | --- |
| **32** | Specify any additional equipment **required** to perform the task or included in regular gear.  *You can check more than one box if necessary.* | Helmet with night vision equipment  Hand-held night vision equipment  Additional weapon(s) (specify which below)  ________________________________  Hand grenades  Protective mask bag with accessories  Life jacket/rescue vest  Radio equipment (specify type below)  _________________________________  Clothing/reinforcement garments for winter conditions  Skis, Snowshoes  Binoculars  Radar/direction-finding equipment  Group equipment (specify below)  ______________________________________  ______________________________________  Aviation duty, additional equipment (specify below)  ______________________________________  ______________________________________  Other equipment (specify below)  ______________________________________  ______________________________________ |

#### 5.5 Frequency at which the physical task is performed in its entirety

|  | Note that the questions in this part (5.5) are about how often (at which frequency) the physical activity is performed **in its entirety.** | |
| --- | --- | --- |
| **33** | With what regularity does the physical task occur **in the military assignment**? | Regularly throughout the entire year  Periodically throughout the entire year  On rare occasions  Perhaps never, but should be able to be performed if the situation demands |
| **34** | How long are the periods during which the physical task is performed **in the military assignment**? | 1–2 days  3–4 days  5–6 days  1–4 weeks  5–8 weeks  9–12 weeks  >12 weeks  The physical task is not performed periodically |
| **35** | How often is the physical task performed **in the military assignment**?  *Specify regardless of whether the task is performed regularly or periodically.* | Daily  5–6 days/week  3–4 days/week  1–2 days/week  2–3 days/week  1 day per month or less |
| **36** | When the physical task is performed **in the military assignment,** how many times per day is it performed?  *Specify regardless of whether the task is performed regularly or periodically.* | 1–5 times/day  6–10 times/day  >10 times/day |
| **37** | With what regularity does the physical task occur **during training** for the military assignment? | Regularly throughout the entire year  Periodically throughout the year  On rare occasions  The task is not included in training for the military assignment |
| **38** | How long are the periods during which the physical task is performed **during training for the military assignment**? | 1–2 days  3–4 days  5–6 days  1–4 weeks  5–8 weeks  9–12 weeks  >12 weeks  The physical task is not performed periodically  The task is not included in training for the military assignment |
| **39** | How often is the physical task performed during **training for the military assignment**?  *Specify regardless of whether the task is performed regularly or periodically.* | Daily  5–6 days/week  3–4 days/week  1–2 days/week  2–3 days/month  1 day per month or less  The task is not included in training for the military assignment |
| **40** | When the physical task is performed **during training for the military assignment,** how many times per day is it performed?  *Specify regardless of whether the task is performed regularly or periodically.* | 1–5 times/day  6–10 times/day  >10 times/day  The task is not included in training for the military assignment |

#### 5.6 Duration for which the physical task is performed in its entirety

|  | Note that the questions in this part (5.6) are about how long (for which duration) the physical activity is performed **in its entirety.** | |
| --- | --- | --- |
| 41 | **How long** is the physical task **usually** performed for **in the military assignment**? | <1 minute  1 minute –15 minutes  >15 minutes – 30 minutes  >30 minutes – 60 minutes  >1 hour – 2 hours  >2 hours – 4 hours  >4 hours – 8 hours  > 8 hours |
| **42** | How long **should** the physical task **be able to be performed for in the military assignment**? | <1 minute  1 minute –15 minutes  >15 minutes – 30 minutes  >30 minutes – 60 minutes  >1 hour – 2 hours  >2 hours – 4 hours  >4 hours – 8 hours  > 8 hours |
| **43** | For how long is the physical task **usually** performed for during **training for the military assignment**? | <1 minute  1 minute –15 minutes  >15 minutes – 30 minutes  >30 minutes – 60 minutes  >1 hour – 2 hours  >2 hours – 4 hours  >4 hours – 8 hours  > 8 hours  The task is not included in training for the military assignment |

#### 5.7 Manual handling

| **44** | **Definitions based on the Swedish Work Environment Authority’s directives:**  Manual handling involves **using the hands** to lift, move, place, hold or carry a load, or to push or pull an object.  *Note: The definition* ***does not include body-worn equipment****, but may encompass activities such as lifting gear or carrying a weapon in the hands as manual handling.* **Lifting (moving/placing)** = manual handling of a load/object that lasts for <5 seconds and occurs without moving position or during movement of <5 metres  **Holding** = manual handling of a load/object that lasts >5 seconds and occurs without moving position or during movement of <5 metres  **Carrying** = manual handling of a load/object during simultaneous movement >5 metres where the load/object **does not have** contact with any surface.  **Push/Pull** = manual handling of a load/object during simultaneous movement where the load/object **does** **have** contact with a surface. | |
| --- | --- | --- |
| **45** | Does the execution of the physical task involve any form of manual handling according to the definitions above? | Yes  No |
|  |  | ***If you have answered “No”, please go to the next part of the protocol that addresses environmental factors (part 5.8).*** |
| **46** | What kind of Manual handling is required for the execution of the physical task?  *You can check more than one box if necessary.* | Lifting (moving/placing)  Holding  Carrying  Pushing  Pulling |
| **47** | Specify the 2 heaviest and/or largest objects that need to be manually handled in the physical task, and estimate their weight and dimensions.    *(example of objects can be weapon, boxes, stretchers, people, etc.)*  *If the task is carried out by more than one person, give the weight handled by each individual e.g. for two people handling an object weighing 50 kg, specify 25 kg.* | Object 1;  _______________________________  Weight (kg);  ________________  Length/Width/Height (cm);  ________/________/________  Object 2;  _______________________________  Weight (kg);  ________________  Length/Width/Height (cm);  ________/________/________ |
| **48** | If you are unable to estimate the weight or dimensions of any object, please explain why. |  |
| **49** | Specify the number of **lifts** that are required **to be able to perform** the physical task. | <5  5 to <10  10 to <20  20 to <40  40 to <60  60 to <80  80 to 100  >100  The physical task does not involve lifting. |
| **50** | How **far should** the object(s) **be able to be carried** during the physical task. | <10 m  10 m to <50 m  50 m to <100m  100 to <250 m  250 m to <500 m  500 m to <1 km  1 km to <5 km  >5km  The physical task does not involve carrying objects |
| **51** | How **far should** the object(s) **be able to be pushed or** **pulled** during the physical task. | <10 m  10 m to <50 m  50 m to <100m  100 to <250 m  250 m to <500 m  >500 m  The physical task does not involve pushing/pulling objects |
| **52** | What **distance** **from the body is the object usually** handled during the performance of the physical task?  *See definitions for lift, hold and carry above.* | If the task involves **lifting** an object;  *Close against the body*  *<30 cm from the body*  *30 cm - 45 cm from the body*  *> 45 cm from the body*  If the task involves **holding** an object;  *Close against the body*  *<30 cm from the body*  *30 cm - 45 cm from the body*  *> 45 cm from the body*  If the task involves **carrying** an object;  *Close against the body*  *<30 cm from the body*  *30 cm - 45 cm from the body*  *> 45 cm from the body*  The physical task does not involve carrying, holding or lifting an object. |
| **53** | How much of the total time the physical task takes involves manual handing (lifting, holding, carrying, pushing, pulling)? | A small part *(<1/3 of the total time)*  A significant part *(1/3–2/3 of the total time)*  A large part *(>2/3 of the total time)* |

5.8 Evironmental factors influencing the execution of the physical task

| **54** | Where is the physical task **most often** performed? | Outdoors  Indoors  On a ship (specify type);  _______________________________  In a vehicle (specify type);  _______________________________  In a plane (specify type);  _______________________________ |
| --- | --- | --- |
| **55** | Where should the physical task **be able to be performed**?  *Check more than one alternative if necessary.* | Outdoors  Indoors  On a ship (specify type);  _______________________________  Ina vehicle (specify type);  _______________________________  In a plane (specify type);  _______________________________ |
| **56** | Does the physical task ever **need to be performed** in a confined space? | No, space is never confined  Yes, space can be confined…  *…low ceiling*  *…restricted area (<1.5 m^2^)*  *…other, please specify;  ___________________________   ___________________________* |
| **57** | What kind of ground/terrain/surroundings is the physical task **usually performed on outdoors**?  *Check more than one alternative if necessary.* | Even (e.g. road, lawn…)  Uneven (e.g. forest, field, meadow...)  Undulating (e.g. mountainous, coastal…)  Swamp  Snow  Ice  In water  Moving surface (e.g. ship, vehicle…)  Other, please specify…  __________________________________   The task is not performed outdoors |
| **58** | What kind of ground/terrain/surroundings **should the physical task be able to be performed on outdoors**?  *Check more than one alternative if necessary.* | Even (e.g. road, lawn…)  Uneven (e.g. forest, field, meadow...)  Undulating (e.g. mountainous, coastal…)  Swamp  Snow  Ice  In water  Moving surface (e.g. ship, vehicle…)  Other, please specify…  __________________________________   The task is not performed outdoors |
| **59** | What temperature is the physical task **usually** performed in?  *Check more than one alternative if necessary.*  *(Degrees Celsius)* | > +30°  +21° to +30°  +11° to +20°  +1° to +10°  ±0°  -1° to -10°  -11° to -20°  -21° to -30°  < -30° C |
| **60** | What is the **highest temperature** that the physical task should be able to be performed in.  *(Degrees Celsius)* | > +30°  +21° to +30°  +11° to +20°  +1° to +10°  ±0° |
| **61** | What is the **lowest temperature** that the physical task should be able to be performed in.  *(Degrees Celsius)* | ±0°  -1° to -10°  -11° to -20°  -21° to -30°  < -30° C |
| **62** | The physical task **should be able to be performed** … | …in the light  …in the dark |
| **63** | Please specify other environmental factors that can hinder the performance of the physical task.  *E.g. strong wind, precipitation, high altitudes, buildings, people etc.* | I have no further suggestions for environmental factors that could hinder the performance of the physical task. |

***Supplementary material 2***

**Proposed and accepted definitions of generic and critical physical job tasks
(in English and Swedish).**

| **English** | |
| --- | --- |
| **Generic physical job task** | A (physical) task within the military assignment that is performed recurrently (daily or several times/week), either on a regular basis or periodically, and that may lead to considerable physical burden to the individual(s) performing the task. |
| **Critical physical job task** | A (physical) task within the military assignment that if unsuccessfully performed could endanger a mission or could be critical to the health and safety of an individual, unit, or the public, or lead to considerable damage of equipment. |
| **Swedish** | |
| **Gränssättande fysisk arbetsuppgift** | En fysisk arbetsuppgift som förväntas genomföras återkommande (flera gånger per vecka eller dagligen), vilket kan ske antingen regelbundet eller periodiskt, i krigsbefattningen och som kan innebära betydande fysisk belastning för individen(erna) som utför uppgiften. |
| **Kritisk gränssättande fysisk arbetsuppgift** | En fysisk arbetsuppgift inom krigsbefattningen där ett misslyckande i utförandet äventyrar uppdraget, alternativt innebär fara för individen, enheten, allmänheten eller leder till väsentlig skada på materiel. |
